# Development of an efficient strategy to improve HPV immunization coverage in Japan

**DOI:** 10.1186/s12889-016-3676-7

**Published:** 2016-09-23

**Authors:** Asami Yagi, Yutaka Ueda, Tomomi Egawa-Takata, Yusuke Tanaka, Akiko Morimoto, Yoshito Terai, Masahide Ohmichi, Tomoyuki Ichimura, Toshiyuki Sumi, Hiromi Murata, Hidetaka Okada, Hidekatsu Nakai, Masaki Mandai, Kiyoshi Yoshino, Tadashi Kimura, Junko Saito, Risa Kudoh, Masayuki Sekine, Takayuki Enomoto, Kei Hirai, Yorihiko Horikoshi, Tetsu Takagi, Kentaro Shimura

**Affiliations:** 1 Department of Obstetrics and Gynecology, Osaka University Graduate School of Medicine, 2-2, Yamadaoka, Suita, Osaka 565-0871 Japan; 2 The Obstetrical Gynecological Society of Osaka, 4-4-3 Kawaramachi, Chuo-ku, Osaka 541-0048 Japan; 3 Department of Obstetrics and Gynecology, Osaka Medical College Graduate School of Medical Sciences, 2-7 Daigakumachi, Takatsuki, Osaka 569-8686 Japan; 4 Department of Obstetrics and Gynecology, Osaka City University Graduate School and Faculty of Medicine, 1-4-3 Asahimachi, Abeno-ku, Osaka 545-8585 Japan; 5 Department of Obstetrics and Gynecology, Kansai Medical University Graduate School of Medicine, 2-3-1 Shin-machi, Hirakata, Osaka 573-1191 Japan; 6 Department of Obstetrics and Gynecology, Kinki University Graduate School of Medical Sciences, 377-2 Ono-Higashi, Sayama, Osaka 589-8511 Japan; 7 Saito Women’s Clinic, 1-6-1 Miyahara, Yodogawa-ku, Osaka 532-0003 Japan; 8 Department of Obstetrics and Gynecology, Niigata University Graduate School of Medical and Dental Sciences, 1-757 Asahimachi-dori, Chuo-ku, Niigata 951-8510 Japan; 9 Institute for Academic Initiatives, Osaka University, 2-2, Yamadaoka, Suita, Osaka 565-0871 Japan

**Keywords:** Adverse events, Behavioural economics, HPV vaccination rate, Internet survey, Governmental recommendation, Decision-making facility, Stepped strategy

## Abstract

**Background:**

In Japan, new HPV immunizations have dropped dramatically after repeated adverse media reports and a June 2013 temporary suspension of the government’s recommendation for the vaccine. The aim of the present study was to develop an efficient strategy to improve HPV immunization coverage across Japan.

**Methods:**

We conducted an internet survey in Japan of mothers of 12–16 year-old girls who were unvaccinated as of May, 2015. The goal was to gather behavioral information from the mothers to develop a strategy for improving Japanese HPV immunization coverage.

**Results:**

Valid survey answers were obtained from 2060 mothers. The survey found that a hypothetical restart of a governmental recommendation for the vaccine would induce 4.1 % of all the mothers surveyed to be more likely to encourage vaccination of their daughters, without any other preconditions. This initial result would be followed by a moderate spread of vaccinations to these daughters’ close friends and acquaintances, hypothetically resulting in a total vaccination rate of 21.0 % of the targeted age-eligible girls. As a second critical step for improving vaccinations, an educational information sheet integrating the concepts of behavioral economics for changing behaviors was found to be significantly effective for persuading mothers with poorer decision-making facilities, who would otherwise prefer to wait to first see the vaccination of other girls of the same age as their daughter.

**Conclusions:**

Following what we foresee as the inevitable restart of the Japanese government’s recommendation for receiving the HPV vaccine, we expect to first see vaccinations occurring in a very small group of girls, the daughters of the most willing mothers, which will be roughly 4 % of those eligible for government paid vaccinations. This will be followed by the spread of vaccinations outward through these girls’ circle of friends and acquaintances, and, finally, to the daughters of the most skeptical mothers, those who would await the return of new vaccine safety results from a large group of similarly-aged girls. As a critical step in improving HPV vaccine coverage in Japan, an educational information sheet that integrates the concepts of behavioral economics for changing behaviors can be employed to persuade mothers with poor decision-making facilities.

## Background

In many developing countries women have a relatively high risk for cervical cancer because efficient and affordable early-detection screening programs for the disease are largely lacking, [1]. In Japan, a developed country with a very high standard of living and Human Development Index, cervical cancer screening is both highly efficient and affordable, yet the country’s screening rate is abysmally low in its youngest at-risk age groups; it is only 10 % for women aged 20–25, and 24 % in those 26–30. For this and other reasons, the incidence of cervical cancer in Japan, especially for women in their twenties and thirties, is rising steadily [2].

In both developing and developed countries, the sexually transmitted human papillomavirus (HPV) is recognized as the leading cause of cervical cancer. HPV contributes to several other cancers as well, i.e., vulvar, vaginal, penile, anal, and oropharyngeal cancers [3]. The preventive efficacy of the HPV vaccine against HPV infection and HPV-associated precancerous lesions is well established [4], indicating that the widest possible dissemination of the vaccine is crucial in reducing the risk for HPV-associated cervical cancer. However, in some countries, and for various reasons, HPV vaccination is not going as well as it should [5–7], which is creating the risk of a disaster.

In Japan, the HPV vaccination rate for age-eligible young girls was as high as 70 ~ 80 % in 2011 and 2012 [8]. By April of 2013, the HPV vaccine program was doing so well that it was added to the government’s list of suggested routine vaccinations, under the Preventive Vaccination Law, which suggested that it be applied to all 12–16 year-old girls (after obtaining an informed consent from their parents or guardians). However, just a few months later, a series of adverse medical events were alleged to have occurred following HPV immunization. By June of 2013, the Japanese Ministry of Health, Labour, and Welfare (MHLW) had temporarily suspended its recommendation for adolescent HPV vaccination [9]. As a direct result of this suspension, the rate of HPV vaccination among adolescent girls in Japan immediately plummeted [8, 10]. By late 2013, the HPV vaccination rate of age-eligible girls in Sakai, Osaka had fallen from the previous year’s 70–80 % to just 3.9 % [10]. By 2014, the rate in Sapporo, Hokkaido had fallen even further, to 0.6 % [10].

In order to better understand the behavioral basis for this dramatic decrease in the HPV vaccination rate in Japan, so as to reverse the fall, we had previously conducted an internet survey in March of 2014 [11]. Our survey results found that a young woman’s acceptance of receiving the HPV vaccine was determined predominantly by her mother’s perceptions of the risks versus the benefits of the vaccine, rather than by the daughter’s own perceptions of it. In our follow-up study, we have conducted a large-scale internet survey of mothers of unvaccinated 12–16 year-old daughters to investigate a more efficient strategy for improving HPV immunization coverage in Japan.

## Methods

The goals of our internet survey included an analysis of the factors that influenced a mothers’ intent to recommend that their daughters receive the HPV vaccination. We looked at a number of different qualifiers under the current anti-HPV-vaccine climate and under a hypothetical situation wherein the government had restarted its recommendation for the vaccine. We looked at several preconditions the mothers might require being fulfilled prior to recommending their daughters’ vaccination. We also examined the efficacy of distributing an educational information sheet concerning cervical cancer, which included information regarding the safety and the efficacy of the HPV vaccine in reducing the risk of cervical cancer. We looked at the effects of this information sheet on the mothers’ intention to inoculate their daughters, and for an association between the mothers’ decision-making facilities and their intent to recommend their daughters’ vaccination.

On May 25th and 26th of 2015, we performed an internet survey of Japanese mothers of 12–16 year-old daughters who had not yet been vaccinated. Before conducting the main survey, 10,000 mothers, listed in an internet survey panel as having such daughters, and as being willing to answer a questionnaire, were pre-screened with questions about their daughters’ age and whether their daughter had yet received the HPV vaccine. If they had two or more daughters, they were asked to answer only in respect to the oldest daughter. The main survey was then distributed by e-mail to 4000 mothers who satisfied these criteria, from which we obtained 2060 valid responses (51.5 %).

The mothers were first asked about their recognition of the optimal time for HPV vaccination for the general population of girls, and about what they considered to be the most practical timing for their own daughters. Next, we investigated what sorts of preconditions the mothers would require be met before recommending their daughters’ HPV vaccination. Based on the results of this initial set of questions, the mothers were then divided into five groups, as follows: Group A: Mothers who would vaccinate their daughters for HPV without any preconditions, even under a continued recommendation suspension (subset Group A-willing), and those who would vaccinate their daughters immediately after a recommendation restart, without other preconditions (subset Group A-restart); Group B: Mothers who would have their daughters vaccinated only after seeing safe inoculation of the daughter’s close friends and acquaintances; Group C: Mothers who would vaccinate their daughters only after seeing the safe inoculation of many other girls of the same general age; Group D: Mothers who would stipulate a range of other preconditions before they would allow their daughters to be vaccinated; Group E: Mothers who wouldn’t encourage inoculation of their daughters under any circumstances. The preconditions, of ‘Vaccinate only after friends and acquaintances have been safely inoculated’, and ‘Vaccinate only after many girls of the same general age have been safely inoculated’, were defined as Preconditions 1 and 2, respectively.

We sought to determine what message content on the information sheet we provided was the most effective at positive motivation, and the characteristics of the target population for whom individual content was most effective. Of the 394 mothers who had responded that their attitude toward the HPV vaccine was favorably altered after reading the educational leaflet, we asked which message points they gave the most weight to concerning helping them make a more favorable decision about their daughters’ HPV vaccination. The answers for each question were scored, and the average score was compared for each group. Scoring was as follows: Very applicable: 2, Applicable: 1, Intermediate: 0, Not applicable: -1. The degree of importance for each information point was evaluated by its mean score. The higher the number, the more importance the mothers gave it when making their hypothetical decision regarding their daughters’ real-world HPV vaccination.

**Fig. 1:**
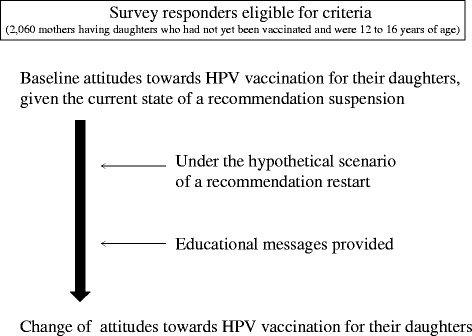
Schema of the survey about mothers’ attitude toward HPV vaccine for their daughters

As part of the survey study, an educational information sheet about cervical cancer and HPV vaccination was presented to the mothers. Its purpose was to evaluate whether educational intervention was effective for increasing the number of mothers who would encourage their daughters to be vaccinated (Fig. 1). To develop a more effective educational information sheet, we used concepts from the field of behavioral economics. Behavioral economics deals with ‘irrational’ economic actors and actions. Previous studies suggested that people often made decisions that deviated from what was expected of ‘rational’ economic actors, and that these decisions were powerfully influenced by their context and reference points [12]. One study showed that using simple posters prompting clinicians to consider their vulnerable patients substantially increased their rates of handwashing [13]. The educational information sheet in the present study was created following a preliminary survey of mothers of 12–16 year-old girls. Mothers used solely for this purpose were not enrolled in the larger internet survey.

### Statistics

**Fig. 2:**
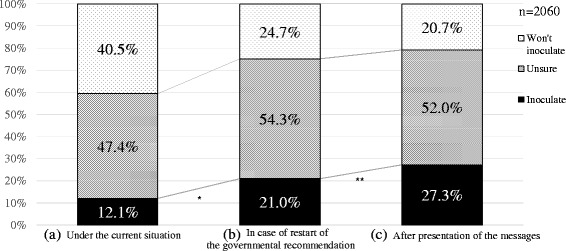
Changes in the mothers’ intention for vaccination of daughter. Shown are the responses of 2060 mothers to a question as to whether they would make their daughter get the HPV vaccine under the current situation (**a**), in case of restart of the govermental recommendation (**b**), and after presentation of the messages (**c**). Spots: will inoculate; Oblique lines: unsure; Black: won’t inoculate. *: The rate of the willing mothers significantly increased after recommendation restart (*p* < 0.001 by Fisher’s exact test). **: The rate of the willing mothers significantly increased after looking at the educational messages (*p* < 0.001 by Fisher’s exact test)

Fisher’s exact test was used to analyze for statistically significant changes in the mothers’ attitudes towards their daughters’ vaccination (Fig. 2). The chi-square test combined with residual analysis was used to investigate the association between the mothers’ attitudes and their decision-making facilities (Table 6). The level of statistical significance was set at *p* = 0.05.

## Results

### Characteristics of the internet survey responders

The relevant characteristics of the survey responders are shown in Table 1. The areas of residence of the responders are distributed fairly consistently with other vital statistic reports for Japan (http://www.ipss.go.jp/syoushika/tohkei/Popular/P_Detail2015.asp?fname=T09-05.htm). The educational background of the respondents was also consistent with the national population census (http://www.stat.go.jp/data/kokusei/2010/index.htm#kekkaga). These facts validate that we had a sufficiently broad representation of study respondents.

**Table 1:** Characteristics of the internet survey responders

	Number	Percent
Age		
30–34	80	3.9
35–39	408	19.8
40–44	933	45.3
45–49	516	25.0
50–54	105	5.1
55–59	14	0.7
Over 60	4	0.2
Residence area		
Hokkaido	99	4.8
Tohoku	136	6.6
Kanto	714	34.7
Chubu	325	15.8
Kinki	429	20.8
Chugoku	106	5.1
Shikoku	51	2.5
Kyushu	200	9.7
Education		
Junior high-school	55	2.7
Senior high-school	657	31.9
Junior or technical college	846	41.1
University	482	23.4
Graduate school	16	0.8
Other	4	0.2

### Attitudes of mothers at the beginning of the survey towards HPV vaccination for their daughters

The initial survey questions pertained to the mothers’ preexisting attitudes towards getting an HPV vaccination for their daughters, given the current state of Japan’s government’s suspension of its recommendation of the vaccine. At this initial point in the survey, only 250 (12.1 %) of the 2060 respondents indicated they were willing to have their daughters vaccinated. The vast majority of responders, 1810 (87.9 %), said they were not currently planning to vaccinate their daughters. Of this larger group, 977 (47.4 %) responded they were unsure or hesitant as to whether or not they were going to recommend their daughter receive the vaccination; the remaining 843 (40.5 %) were clearly unwilling to recommend the vaccination (Fig. 2(a)).

### Recognition of optimal vaccination timing for girls in general and for their own daughter

Of the 1810 mothers who stated at the beginning of the survey that they were not currently planning to vaccinate their daughters, we inquired about their recognition of the optimal timing for the general population of adolescents to receive the HPV vaccination, and about what they considered to be a realistic timing for their own daughter’s vaccination. Even among those mothers who were initially strongly opposed to the HPV vaccine, more than half (57.6 %) correctly recognized that the most effective timing for HPV vaccination was when given *before* sexual debut, whereas only 2.4 % incorrectly thought that giving the vaccination just before pregnancy was the most effective timing (Table 2). However, almost half of these knowledgeable mothers (47.3 %) still intended to put off the vaccination of their own daughters. Reasons given were: 36.8 % would not get their daughters vaccinated until they became an adult; and 10.5 % thought they should wait until the first pregnancy (Table 2).

**Table 2:** Best timing for administration of HPV vaccine

	Number	Percent
The most appropriate inoculation time of HPV vaccination is?		
Before first sexual intercourse	1042	57.6
Don’t know	596	32.9
None in particular (anytime)	117	6.4
Before pregnancy or delivery	43	2.4
Other	12	0.7
Total	1810	100.0
When will your daughter’s HPV vaccination might actually occur?		
Before pregnancy or delivery	109	10.5
Before becoming an adult	383	36.8
Eighteen	115	14.9
Seventeen	99	9.5
Sixteen	18	1.7
Fifteen	110	10.6
Fourteen	9	0.9
Thirteen	10	1.0
Other	149	14.3
Total	1042	100.0

What is ‘the most appropriate inoculation time for HPV vaccination’ was asked of 1810 mothers who, at the beginning of the study, were not willing to vaccinate their daughters. The timing ‘When HPV vaccination might actually occur’for their own daughters was asked of those who correctly answered “Before first sexual intercourse” to the above question

### Under a hypothetical scenario of a restart of the governmental recommendation, changes in mothers’ attitude towards daughters’ vaccination

Among the 2060 responding mothers, when given a hypothetical scenario of ‘a recommendation restart’, 433 (21.0 %) became obviously more willing to get their daughters vaccinated. On the other hand, 1627 (79.0 %) remained skeptical or were otherwise still unwilling to vaccinate their daughters (Fig. 2(b)). We found that the number of willing mothers was significantly increased under the hypothetical recommendation restart scenario (*p* < 0.001, by Fisher’s exact test).

### Preconditions mothers would require to happen before seeking their daughters’ HPV vaccination

We investigated the other preconditions some of the mothers might require be met before they would encourage their daughters’ HPV vaccination. In the group of 250 willing mothers, only 2.0 % (five) answered that they would vaccinate their daughters without any specific preconditions occurring first (Table 3). 26.4 % (66) answered that they would vaccinate their daughters only after observing the inoculation of friends or acquaintances of their daughter (set as precondition 1), whereas almost twice as many mothers, 47.6 % (1.8 times, 119), answered that they would vaccinate their daughters only after witnessing the safe vaccination of many girls of the same age group as their daughter (set as precondition 2). In other words, almost half the women thought that a recommendation restart combined with evidence of a safe vaccination of those closest to them was still insufficient for motivating them to change their minds and recommend the vaccination to their daughter. These results implied that even willing mothers usually stipulated some preconditions before they would recommend their daughters’ inoculation.

**Table 3:** Preconditions mothers required for their daughters’ HPV vaccination

Intention for inoculation				
	Willing	Unsure/Won’t		
		In the case of restart of the governmental recommendation		
		Inoculate	Unsure	Won’t inoculate
Preconditions				
Vaccinate without any specific conditions under suspension of the recommendation (Group A-willing)				
	5/250 (2.0 %)	―	―	―
Vaccinate immediately after restart of the recommendation (Group A-restart)				
	47/250 (18.8 %)	33/183 (18.0 %)	―	―
Vaccinate after friends or acquaintances have been inoculated (Precondition 1)				
(Group B)				
	66/250 (26.4 %)	53/183 (29.0 %)	210/1118 (18.8 %)	19/509 (3.7 %)
Vaccinate after many girls of same age group have been inoculated (Precondition 2)				
(Group C)				
	119/250 (47.6 %)	92/183 (50.3 %)	709/1118 (63.4 %)	126/509 (24.7 %)
Others				
(Group D)				
	13/250 (5.2 %)	5/183 (2.7 %)	199/1118 (17.8 %)	45/509 (8.8 %)
Won’t inoculate				
(Group E)				
	―	―	―	319/509 (62.8 %)
Total				
	250	183	1118	509

Group A: Mothers who would vaccinate their daughters without preconditions, even under recommendation suspension (Group A-willing), and those who would vaccinate their daughters immediately after a recommendation restart, without other preconditions (Group A-restart)

Group B: Mothers who would vaccinate their daughters only after inoculation of close friends and acquaintances

Group C: Mothers who would vaccinate their daughters only after inoculation of many other girls of the same age group

Group D: Mothers who would stipulate other preconditions before they will vaccinate their daughters

Group E: Mothers who wouldn’t inoculate their daughters under any circumstances

Next, we analyzed the survey responses from the 1810 mothers who answered that their intentions were uncertain or that they were unwilling to vaccinate their daughters under the current suspension of governmental recommendation. Among those who were willing to inoculate their daughter under the hypothetical scenario of a recommendation restart, the ratio of mothers having a strict requirement for precondition 2 versus 1 was 1.7 (50.3 to 29.0 %). Among those who were unsure whether to vaccinate even under this scenario, the ratio was 3.4 (63.4 to 18.8 %). Among those who decided they would not inoculate their daughters, even under the scenario of a recommendation restart, the ratio was 6.6 (24.7 to 3.7 %). These results imply that the preconditions mothers might require prior their daughters’ inoculation were well correlated with their positive or negative intentions for the subsequent vaccination of their daughters.

Based upon their responses to the hypothetical restart scenario, we divided the mothers into five groups according to the preconditions they stipulated, as shown in Table 3. Of all mothers surveyed, the percentage of Group A mothers who intended to vaccinate their daughters without any pre-requirements was only 4.1 % (5 + 47 + 33 = 85, out of 2060). The percentage of Group B was 16.9 % of the total (66 + 53 + 210 + 19 = 348, out of 2060), resulting in a total ‘hypothetical vaccination rate’ after a ‘hypothetical recommendation restart’ of 21.0 % (5 + 47 + 33 + 66 + 53 + 210 + 19 = 433/2060). The percentage of Group C was 50.8 % (119 + 92 + 709 + 126 = 1046/2060). Groups A, B, and C, which were thought to be immediate targets for induction of their daughters’ vaccination after restart of the governmental recommendation, accounted for 71.8 % of all mothers surveyed.

### Effect of behavioral economics-based messages provided to mothers to promote desired decision-making towards the daughters’ HPV vaccination

Utilizing perspectives gained from the field of behavioral economics, we designed five message sheets to promote the mothers making a decision in favor of their daughters’ HPV vaccination, as shown in Table 4. After the educational messages were presented to them in the mid-point of the survey form, we explored changes in the mothers’ attitude towards their daughters’ HPV vaccination, continuing with the scenario of a hypothetical recommendation restart. Before reading the message sheets, 27.3 % of the mothers (562/2060) had said they would be willing to direct that their daughters receive the HPV vaccine. After reading the educational messages, the percentage of mothers willing to recommend the vaccine increased significantly, from 21.0 to 27.3 % (*p* < 0.001, by Fisher’s exact test, Fig. 2(c)), suggesting some efficacy for the messages for attitude-change.

**Table 4:** Educational messages

Content of the messages
Message 1: Loss of future fertility by not vaccinating
-Invasive cervical cancer in younger women has doubled, compared to 20 years ago
-Hysterectomy is usually needed for invasive cervical cancer, even if it is found at an early stage, and afterwards the patient can’t conceive
Message 2: Loss of the future benefits by postponement of vaccination
-Efficacy of HPV vaccine is reduced if girl not inoculated before first sexual intercourse
Message 3: Expectations for the vaccine (dispelling the negative image of adverse events)
-The Nobel Prize was awarded for discovery of HPV
-The vaccine has been developed through study for as long as 20 years after discovery of HPV
-The vaccine has been approved in about 120 countries/regions worldwide
Message 4: Comparison of future benefits by vaccination with near-term losses
-The vaccine is effective in preventing cervical cancer, with a probability of 60–70 % in the future
-Severe adverse events occur after vaccination in only about 0.007 % of girls
Message 5: Safety of vaccination (changing the focus away from serious adverse events)
-HPV vaccine is given safely in about 99.993 % of girls

Message 1 was created using the theory of ‘loss avoidance’, which is one of the most representative theories in behavioral economics. By emphasizing the increase of cervical cancer incidence in younger women compared to 20 years ago, and the possibility of needing to undergo a hysterectomy for cervical cancer, Message 1 explains the magnitude of loss caused by avoiding vaccination. Conservative mothers usually attempt to minimize change. Message 2 aimed to make conservative mothers recognize future loss caused by postponement of vaccination; it told them that selecting the conservative status quo meant selecting the possibility of their daughter suffering cervical cancer [2]. Message 3 conveyed information to increase confidence in the reliability and safety of the vaccine, because many of the mothers obviously felt uneasy about it. This message encouraged the mothers to perceive of the vaccination more positively by dispelling the near-term-loss image of adverse events. Message 4 used a numeric comparison of the merits and risks of vaccination. It showed how unwillingness to vaccinate their daughters meant selection of future bad consequences for them. For creating Message 5, we utilized the framing theory of behavioral economics, by creating a way to show numerical data that significantly affects decision-making. In this message, the safety of the HPV vaccine was expressed by the impressive-looking number of 99.993 %, thus changing the focus from the rare probability of severe adverse events, 0.007 % [14]

### Message elements that aided deciding to seek daughters’ vaccination

“Evaluation of safety by experts”, “Recommendation from a doctor” and “Recommendation from the national and local governments” (scores of 1.44, 1.19, and 0.91, respectively) were regarded as most important message elements by mothers in Group A, whereas these three messages had a lower range of importance in the other groups (0.90–1.41, 0.24–1.14, and 0.07–0.88, respectively) (Table 5). In Group B, the “Opinion of her husband or family” and “Opinion of person close to her” were more critical than for mothers in the other groups. In the same way, detailed information about adverse events, such as “Clinical outcome of the cases with the adverse events” and “Frequency of adverse events” were more important for motivating those mothers in Groups C and D than in Groups A and B.

**Table 5:**
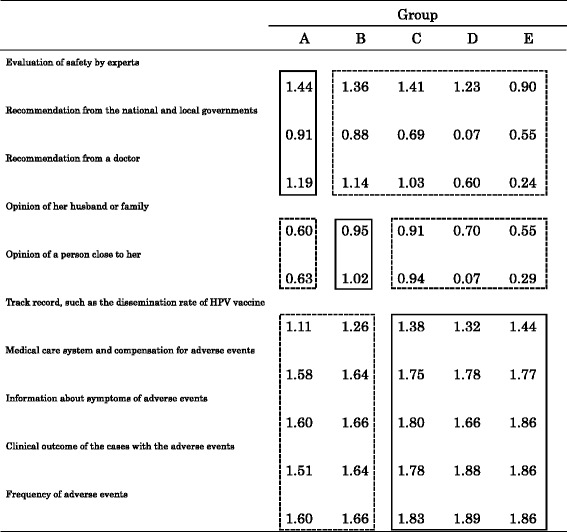
Degree of importance the mothers rated the message points when making a decision concerning their daughters’ actual HPV vaccination

Group A: Mothers who would vaccinate their daughters without preconditions, even under recommendation suspension (Group A-willing), and those who would vaccinate their daughters immediately after a recommendation restart, without other preconditions (Group A-restart) Group B: Mothers who would vaccinate their daughters only after inoculation of close friends and acquaintances Group C: Mothers who would vaccinate their daughters only after inoculation of many other girls of the same age group Group D: Mothers who would stipulate other preconditions before they will vaccinate their daughters Group E: Mothers who wouldn’t inoculate their daughters under any circumstances The answers for each question were scored and the average score was compared for each group. Scoring was as follows: Very applicable: 2, Applicable: 1, Intermediate: 0, Inapplicable: -1, Not applicable at all: -2

These results imply that just receiving a recommendation letter from the national or local government might be sufficient to induce many mothers in Group A to vaccinate their daughters, whereas highly focused informative messages were required to make the mothers in Groups C, D, and E change their mind and inoculate their daughters. In each group of mothers there appears to be a different way needed to change their attitudes toward HPV vaccination.

### Decision-making facility of the mothers and their attitude towards their daughters’ HPV vaccination

The association between the decision-making facility of the mother and her attitude toward her daughters’ HPV vaccination was investigated next. We evaluated the mother’s decision-making facility by asking whether or not she usually felt it was difficult to make decisions in her daily life. The mothers who felt they were able to make decisions easily were classified into a high decision-making facility group, and those who were not able to make decisions easily were classified into a low decision-making facility group. The survey found that the mothers’ attitudes towards HPV vaccination, under the current situation of recommendation suspension, was not related to their decision-making facility (*p* = 0.14, by the chi-square test) (Table 6). However, their ability to change their attitudes under the scenario of a recommendation restart did tend to be associated with their decision-making facility (*p* = 0.065, by chi-square test).

**Table 6:** Decision-making facility of mothers and their attitude towards their daughters’ HPV vaccination

Intention of inoculation				
	Willing	Unsure	Won’t	*p*-value
	*n* (%)	*n* (%)	*n* (%)	(chi-square test)
As the current situation				
Decision-making facility				
High	160 (13 %)	566 (46 %)	510 (41 %)	0.14
Low	90 (11 %)	411 (50 %)	323 (39 %)	
In the case of a restart of the governmental recommendation				
Decision-making facility				
High	262 (21 %)	648 (52 %)	326 (26 %)	0.065
Low	171 (12 %)	470 (57 %)	183 (22 %)	
After educational intervention (messages presented)				
Decision-making facility				
High	326 (26 %)	627 (51 %)	283 (23 %)^a^	0.012
Low	236 (29 %)	444 (41 %)	144 (17 %)^a^	

^a^ *p* < 0.05 by chi-square test combined with residual analysis

Those having the highest decision-making facility also initially tended to be the most stubbornly resistant to changing their minds towards inoculating their daughters following just the restart scenario, and even more so after reading the information sheets. The shift in group numbers, from unwilling, to unsure, to willing, could be seen.

Most interestingly, after presentation of the educational information, the mothers who had a lower decision-making facility tended to be the ones who were most positively influenced by the educational material. The chi-square test combined with residual analysis revealed that, among those who were initially unwilling to inoculate their daughters, the ratio of the number of the mothers of high decision-making facility versus the number of those of low decision-making facility was significantly higher (283/144) than among those who were unsure (627/444), and those who were willing (326/236) (*p* < 0.05). This strongly indicates an educational intervention might be a more effective means to induce those with a low decision-making facility to decide to inoculate their daughters.

## Discussion

The goals of the present study were to survey Japanese mothers about their intentions regarding recommending that their daughters’ receive an HPV vaccination, and to examine how those intentions might best be positively changed. Our previous internet survey suggested that a young woman’s acceptance of the HPV vaccine, under the current anti-vaccine climate in Japan, is determined predominantly by her mother’s perceptions concerning the vaccine [11]. A mere listing of dry facts about cervical cancer and the safety of the HPV vaccine has been shown to be largely ineffective for changing a mothers’ attitude.

In the present analysis, we have demonstrated that, in general, Japanese mothers tend to put off their daughters’ vaccination, even when they understood that the most appropriate inoculation timing was during their early teenage years, before sexual activity usually begins (Table 2). Our survey found that the less willing the mothers were to seek their daughters’ vaccination, the more stringent were the preconditions they stipulated before they would change their mind (Table 3). We also demonstrated that it will be extremely difficult to improve vaccination rates because of the negative images about the HPV vaccine indoctrinated into the mothers’ minds by the extensive reporting, by both regular and social media, of the alleged severe adverse post-vaccine events, and by the government’s suspension of its own recommendation for receiving the HPV vaccine. Today, 3 years and counting after the government announced its recommendation suspension, only 12.1 % of the mothers responded that they are willing to make their daughters receive the HPV vaccine (Fig. 2(a)). Moreover, even among those mothers with positive attitudes towards the vaccine, the number who answered they were willing to recommend their daughters get vaccinated, without any specific preconditions being met, was only 2.0 % (Table 3). Of all 2060 responding mothers, an infinitesimal 0.24 % were openly willing to recommend vaccination, corresponding roughly to the actual current rate of new HPV vaccinations occurring in Japan ([8], and our unpublished data).

Most mothers, even those with an initial positive appreciation toward the HPV vaccine, stipulated some preconditions for their daughters’ HPV vaccination. Not surprisingly, these stipulated preconditions became stricter in parallel with the mothers’ initial degree of negative intentions to make their daughters get vaccinated. The percentage of Group B (acquaintances first) mothers was 6.6 times as high as those in Group C (many other girls in same age group), whereas that was 1.8 times Group A–willing-regardless, 1.7 times the Group A-after-restart, and 3.4 times the Group C-unsures.

The percentage of Group A mothers who intended to vaccinate their daughters without any preconditions was only 4.1 % of all mothers (85 / 2060). The percentage of Group B mothers was 16.9 % of the total (348/2060), resulting in a total ‘hypothetical vaccination rate after a hypothetical recommendation restart’ of 21.0 % (433/2060). Group C represented 50.8 % of all mothers (1046 / 2060), resulting in a hypothetical best case total vaccination rate of 71.8 %.

These findings are supported in a different way by the results shown in Table 5. A recommendation for the HPV vaccine from authorities, including doctors, experts, or the government, was sufficient to convince the Group A mothers (those who intended to make their daughters vaccinate under the current recommendation suspension or soon after a recommendation restart) to make a decision in favor of their daughters’ vaccination; whereas more detailed information about the adverse events was shown to be required for mothers who stipulated more strict preconditions before undertaking inoculation, or who otherwise would not vaccinate their daughters (Groups C, D and E).

Next, we analyzed several educational messages used to attempt to induce the mothers to make their daughters vaccinate. Five educational messages were created utilizing the principles of a sub-field of behavioral science known as behavioral economics, which is the study of the effects of psychological, social, cognitive, and emotional factors on the economic decisions of individuals and their consequences for market prices, values, returns, and resource allocation. Behavioral economics explores why people sometimes make irrational decisions, and why and how their behavior does not always follow the predictions of economic or medical health models.

In our previous study [12], we found that a mere re-statement of the dry facts concerning cervical cancer and the HPV vaccine, including its safety and efficacy, was largely ineffective in changing a reluctant mother’s attitudes. For the current study, we developed and presented five redesigned message sheets, using behavioral economics guidelines to guide the wording. These new sheets were found to be significantly more effective at increasing the numbers of mothers who intended to make their daughters vaccinate (*p* < 0.001) (Fig. 2 (c)).

The decision-making facility of the mothers, and how that trait related to their attitude toward their daughters’ HPV vaccination, was further investigated. Interestingly, the mother’s attitude of whether or not to make their daughters vaccinate under the current recommendation suspension was not associated with their decision-making facility (*p* = 0.14). The mothers who felt they had a high decision-making facility tended significantly to not want to inoculate their daughters, whereas those of low decision-making facility (indecisive) tended to be easily influenced to inoculate their daughters by reading the educational messages. This suggests that the educational intervention approach might be the most effective for inducing those of low decision-making facility to decide to inoculate their daughters.

Based on our latest survey information, and given the current reality of a lack of governmental support, we can now ask: How can we utilize the message sheet method to most effectively persuade mothers to decide in favor of HPV vaccination for their daughters? We accept that it is virtually impossible to survey everybody, or to get completely honest responses, in order to then target individualized messages to mothers’ based in advance on their answers to survey questions about their present attitudes toward the HPV vaccine, or the preconditions which they might stipulate for inoculation of their daughters, or their decision-making facility.

Based on the results of the present study, we propose that a stepped strategy might work. For the willing mothers, who would fall into Groups A and B, a well-publicized official announcement of the restart of recommendation, or even declarative statements by medical experts about the vaccine’s safety, will be sufficient; for them, additional intervention should not be required. In the case of a recommendation restart, these mothers will be the first to start inoculation of their daughters, resulting in a vaccination rate of roughly 4 %, possibly within a few months from a recommendation restart. These girls’ friends and acquaintances will be reassured about the vaccine’s safety and will feel peer pressure to receive the HPV vaccination soon after, without any additional intervention, because their mothers tend to put great value on the opinions of their family and friends (Table 3). This second wave should result in a vaccination rate approaching 21.0 %.

However, the next steps will be painfully incremental. There will be roughly 50 % of the mothers who will not vaccinate their daughters until they see that many other girls in the same generation as their daughters have been safely vaccinated first. These mothers will likely stipulate a requirement to be provided with detailed information about any potential for new adverse events that might occur, no matter how unlikely or inconsequential. For this group of hard sales, an educational sheet providing safety information utilizing behavioral economics to guide the wording might be effectively used.

In the present study, the mothers who would stipulate a requirement for vaccination of many girls in the same generation as their daughters, irrespective of the governmental recommendation status, were the largest sub-population in our study (1046 out of 2060; 50.8 %) (Table 3). If this population could be persuaded to vaccinate, the coverage rate might approach 70 %. In such a situation, many mothers will feel peer pressure, that everyone else is safely receiving the HPV vaccination, and it will influence them.

Our study has its limitations. In the internet survey, the mothers answered questions regarding their intention to vaccinate their daughters under hypothetical situations. The results might not necessarily predict the vaccination rate under real situations. Additionally, although the net effect of the educational information we provided did increase the mothers’ intention for inoculation, it improved only to 27.3 %, and this rate might not necessarily fulfill the precondition requirement for a recognition that ‘many girls of the same age group have been safely inoculated’. Further refinements in the types, amounts, timing and delivery systems for these educational tools will be required to overcome this gap.

## Conclusions

In conclusion, our survey found that, due to the current climate of fear and uncertainty surrounding the HPV vaccine, Japanese mothers are tending to put off their daughters’ HPV vaccination, even when they understand and accept its benefits. It is going to be highly difficult to induce these mothers to inoculate their daughters, even after the eventual restart of the governmental recommendation, because many of these mothers intend to wait for the vaccination of many other girls before they will commit their own daughters. For the most effective dissemination of the HPV vaccine after a recommendation restart, vaccination of the daughters of the most willing mothers will have to occur first, and the hope is that this will spread to their friends and acquaintances.

Our survey of responses to our educational sheet, which was developed and guided by behavioral economics, demonstrated that it functioned effectively to persuade mothers of low decision-making facility to want to have their daughters vaccinated within a few months of a recommendation restart (Table 6). During these months, educational leaflets should be utilized to reinforce this motivation. This stepped strategy should be practiced in small scale trials in anticipation of a restart of the governmental recommendation.

It is hoped that the present study might provide a useful HPV educational tool, not only for Japan, but also for the many other countries where the HPV vaccination rate is woefully insufficient.

## Abbreviations

HPV: Human papillomavirus
MHLW: The Japanese Ministry of Health, Labour, and Welfare
